# Identification of *Cdk8* and *Cdkn2d* as New Prame-Target Genes in 2C-like Embryonic Stem Cells

**DOI:** 10.3390/genes13101745

**Published:** 2022-09-27

**Authors:** Valeria Lucci, Elena De Marino, Daniela Tagliaferri, Stefano Amente, Alessandra Pollice, Viola Calabrò, Maria Vivo, Geppino Falco, Tiziana Angrisano

**Affiliations:** 1 Department of Biology, University of Naples “Federico II”, 80147 Naples, Italy; 2 IEOS-CNR, Institute of Experimental Endocrinology and Oncology “G. Salvatore”—National Research Council, 80131 Naples, Italy; 3 Biogem Scarl, Istituto di Ricerche Genetiche “Gaetano Salvatore”, 83031 Ariano Irpino, Italy; 4 Department of Molecular Medicine and Medical Biotechnology, University of Naples “Federico II”, 80131 Naples, Italy; 5 Department of Chemistry and Biology, University of Salerno, 84084 Fisciano, Italy

**Keywords:** PRAME, embryo stem cell, RA-resistant

## Abstract

Embryonic stem cells (ESCs) present a characteristic pluripotency heterogeneity correspondent to specific metastates. We recently demonstrated that retinoic acid (RA) induces an increase in a specific 2C-like metastate marked by target genes specific to the two-cell embryo stage in preimplantation. Prame (Preferentially expressed antigen in melanoma) is one of the principal actors of the pluripotency stage with a specific role in RA responsiveness. Additionally, PRAME is overexpressed in a variety of cancers, but its molecular functions are poorly understood. To further investigate Prame’s downstream targets, we used a chromatin immunoprecipitation sequencing (ChIP-seq) assay in RA-enriched 2C-like metastates and identified two specific target genes, *Cdk8* and *Cdkn2d*, bound by Prame. These two targets, involved in cancer dedifferentiation and pluripotency, have been further validated in RA-resistant ESCs. Here, we observed for the first time that Prame controls the *Cdk8* and *Cdkn2d* genes in ESCs after RA treatment, shedding light on the regulatory network behind the establishment of naïve pluripotency.

## 1. Introduction

Prame, *Gm12794c* in mice, is a member of a multigene family present in humans and other mammals [1]; it is expressed in different cancers [2] and is associated with a novel stem cell molecular signature in the 2C-like metastate, characteristic of naïve pluripotency [3]. ESCs are characterized by different metastable subpopulations that fluctuate among different levels of pluripotency, defined as metastates. These metastates are marked by specific factors whose expression affects the state of cell pluripotency [4,5,6]. Occasionally, a metastable subpopulation may convert to a highly pluripotent metastate (2C-like) resembling the two-cell stage (2C) embryos, in which a rearrangement of chromatin induces a reprogramming of a transcription named zygotic genome activation (ZGA) [7].

Recently, it was demonstrated that RA enhances ESCs’ metastate subpopulation, named Zscan4 metastate, marked by a specific 2C-like gene signature, including *Prame* and *Zscan4,* in which ESCs retain both self-renewal and pluripotency capability [8,9,10]. Correlated to this aspect, human PRAME overexpression has been demonstrated to block retinoic acid (RA)-mediated cell differentiation, cell growth arrest, and apoptotic death, suggesting that PRAME acts as an inhibitor of the retinoic acid receptor (RAR) [11,12,13]. Moreover, PRAME overexpression was found to promote leukemic cell proliferation and inhibit all-trans retinoic acid (ATRA)-induced myeloid differentiation [14] and in several hematological malignancies characterized by the block of myeloid differentiation [15]. Indeed, the murine Prame counteracts RA-dependent differentiation. RA induces high levels of Prame, contributing to the overall DNA hypomethylation and global increase in H3K27 acetylation levels throughout the *Cdkn1a* transcriptional repression induced by the PCR2 complex [13]. The considerable heterogeneity among the different Prame isotypes made it challenging to determine their specific function, so the clinical relevance of Prame remains still unclear and prompted us to investigate whether Prame is a suitable ATRA-resistance therapeutic target. To better understand this aspect, we performed a Prame chromatin immunoprecipitation followed by sequencing (ChIP-seq). We found Prame-specific binding to *Cdk8* and *Cdkn2d* regulatory regions by this approach. We focused our attention on these two targets because of their involvement in cancer dedifferentiation and pluripotency.

Cdk8 is a cyclin-dependent kinase implicated in cellular homeostasis and developmental programming, and it has been shown to regulate several signaling pathways that are crucial regulators of both embryonic stem cell pluripotency and cancer [16].

On the other hand, Cdkn2d is a member of the INK4 family of cyclin-dependent kinase inhibitors that generally regulate the G1-to-S phase transition [17]; interestingly, *Cdkn2d* dissymmetric mRNA distribution was observed in the 2-cell stage in blastomeres [18].

Our ChIP-seq data were validated by chromatin immunoprecipitation and gene expression analysis in ES*^pZscan4^*^-EME/Prame-FLAG^ transgenic cell line. Moreover, we used a second transgenic cell line, overexpressing Prame, to analyze the Prame -specific activity on *Cdk8* and *Cdkn2d* gene expression to maintain the pluripotent metastate in cells treated or not treated with RA.

## 2. Materials and Methods

### 2.1. Cell Culture

The pZscan4-Emerald cells, a gift from Dr. Minoru S.H.Ko, were transfected with the Prame-3xFlag vector (ES*^pZscan4^*^-EME/Prame-FLAG^), generated in [13], and validated for pluripotency expression markers after RA treatment. It was necessary to use the FLAG-tag for Prame immunoprecipitation analysis as there is no available antibody against the murine isoform of the Prame studied.

ES*^pZscan4^*^-EME/Prame-FLAG^ cells were cultured in gelatin-coated 6-well plates in a complete ES medium: DMEM (Sigma-Aldrich, St. Louis, MO, USA); 15% FBS (EuroClone, Italy); 1000 U/mL leukemia inhibitory factor (LIF) (ESGRO, Chemicon, SIGMA, Darmstad, Germany); 1 mM sodium pyruvate; 0.1 mM nonessential amino acids (NEAA), 2.0 mM l-glutamine (Invitrogen, Waltham, MA, USA), 0.1 mM β-mercaptoethanol, and 500 U/mL penicillin/streptomycin, 125 μg/mL G418, and 2.5 μg/mL Blasticidin (Sigma-Aldrich). ESCs were incubated at 37 °C with 5% CO_2_. To enrich Zscan4-EME positive cells (EME+) expressing Emerald protein, the ES*^pZscan4^*^-EME/PRAME-FLAG^ cells were treated with 1.5 μM RA for 4 days. EME+/NoFLAG, containing only *Zscan4* promoter fuser to Emerald protein, was used as a negative control in the immunoprecipitation assay with ant-FLAG antibodies.

The second ES cell line Prame inducible by tetracycline, ES^Gm12794cp2Lox^ was initially described and validated in [13], and in this study it is named ES^Pramep2Lox^. Briefly, the coding sequence of *Prame* was amplified from an available plasmid and cloned into a p2Lox targeting vector. Cellular clones were grown with 275 μg/mL neomycin (Invitrogen), and an empty p2Lox vector stably transfected in ESCs was used as a negative control cell line.

ES^Pramep2Lox^ and p2Lox empty vector cell lines were cultured in DMEM (Invitrogen) supplemented with 15% ES-certified FBS (Invitrogen), 0.1 mM nonessential amino acids (Invitrogen), 1 mM sodium pyruvate (Invitrogen), 0.1 mM β-mercaptoethanol (Sigma-Aldrich), 50 U mL^−1^ penicillin/50 μg mL^−1^ streptomycin (Invitrogen), and 1000 U mL^−1^ LIF (Sigma-Aldrich). Clones were treated for 72 h in the absence or presence of 1.5 μg/mL doxycycline to induce transgene expression, followed by the presence or absence of RA 1.5 μM for 4 days.

Finally, an E14 ES transient knockdown of the *Prame* gene was generated. The *Prame* shRNA was transfected into a pLKO.1 vector (Addgene, Arsenal, UK) using AgeI and EcoRI restriction enzymes. All these passages were verified by sequence analysis. The ES cells were transfected with Lipofectamine Transfection Reagent (Invitrogen^TM^) according to the manufacturer’s instructions.

### 2.2. Flow Cytometry and Sorting of ESpZscan4-Em/Prame-FLAG Transgenic Cell Lines

1 × 10^6^ cell line ES*^pZscan4^*^-EME/Prame-FLAG^ was plated in p100 and treated with 1.5 μM all-trans RA for 4 days and the medium was changed every day. ES E14 cell lines, plated in ES medium and treated with RA, were used as a negative control.

Then, the cells were harvested by Trypsin (Invitrogen) and resuspended in a complete ES medium containing 25 mM HEPES buffer and by FACS-sorted according to the fluorescent intensity of Emerald into a complete ES medium containing HEPES and separated in EME+ (Zscan4/Emerald expressing) and EME-(Zscan4/Emerald not expressing) as performed in [19].

Cell sorting experiments were performed by the cell sorter FACSAria and analyzed through FACSDiva Software Version 6.1.3 (Becton Dickinson, Franklin Lakes, NJ, USA). Analysis of forward scatter (FSC) vs. side scatter (SSC) dot plots excluded dead cells and debris. Afterward, an FSC-Area vs. FSC-Height dot plot was used to identify single cells and exclude doublets. In all experiments, purity was higher than 95% of the desired cells.

### 2.3. Western Blot Analysis

Total proteins extracted from EME+ and EME− cells were analyzed by western blot as described in [20]. The antibodies used are as follows: mouse monoclonal antibody anti-FLAG M2 (Sigma Aldrich, Milan, Italy) and rabbit polyclonal anti-Gapdh (Santa Cruz Biotechnology, Heidelberg, Germany).

### 2.4. qPCR Analysis

For qPCR analysis of FACS-sorted cells, total RNA was collected immediately after sorting from EME+ and EME- cells by TRIzol (Invitrogen) as performed in [21]. The integrity of the RNA was assessed by denaturing agarose gel electrophoresis (presence of sharp 28S, 18S, and 5S bands) and spectrophotometry. A total of 1 μg of RNA of each sample was reverse transcribed with QuantiTect^®^ Reverse Transcription (Qiagen, Germany, Europe) according to the manufacturer’s instructions. qPCR analyses were performed using 20 ng cDNA per well in duplicate with SYBR green master mix (Applied Biosystems, Waltham, MA, USA) according to the manufacturer’s instructions. Reactions were run on Applied Biosystem 7500 (Applied Biosystems). Fold induction was calculated and normalized with the DDCt method and considered the values of at least three independent experiments. The gene-specific primers are available in Table 1.

### 2.5. ChIP Analysis

ChIP analysis was performed as previously described [22] using the EpiQuikTM chromatin immunoprecipitation kit from Epigentek Group Inc. (Brooklyn, NY, USA). Then, 2 × 10^6^ cells sorted into EME+ and EME- were cross-linked in a fresh culture medium with 1% formaldehyde for 10 min, sonicated and used for each immunoprecipitation with 3 μg of antibodies. Immunoprecipitated DNA was used for massively parallel sequencing or analyzed on a real-time PCR machine (Applied Biosystem 7500), SYBR green master mix according to the manufacturer’s instructions. Primer sequences are listed in Table 1.

### 2.6. ChIP-Sequencing, Mapping, and Peak Analysis

Prame immunoprecipitation was used as anti-FLAG antibody on cells EME+ and EME- using independent biological replicates. Single-end 50-bp libraries were prepared from Genomix4life.

**Figure 1:**
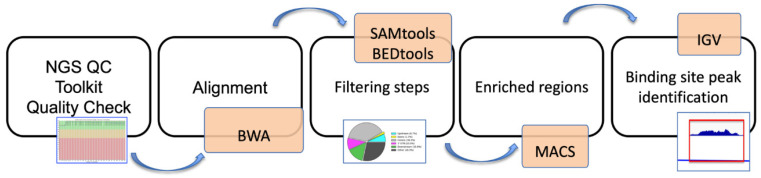
**Flow chart for ChIP-seq analysis in RA-treated EME+ cells.** The multiple steps involved in the analyses of ChIP_seq data are schematically displayed.

The signal obtained by precipitation with the control EME- and input was subtracted from the signals obtained with the specific antibodies. Results are expressed as a percentage of the input, and calculations considered the values of at least two biological replicates. ChIP-seq libraries were prepared with 10 ng of ChIP (or Input) DNA with TruSeq ChIP Sample Prep Kit according to the manufacturer’s instructions of Illumina sequencing. Before sequencing, libraries were quantified using Qubit (Invitrogen) and quality-controlled using Agilent’s bioanalyzer. The basic steps are represented in the form of a flowchart in Figure 1.

A 50 bp single-end sequencing was performed using Illumina HiSeq 2000 platform (Genomix4life S.R.L., Baronissi, Salerno, Italy) according to standard operating procedures. ChIP-seq reads were quality-checked with NGS QC Toolkit. Alignments were performed with BWA to the reference genome mm9 (mouse assembly July 2007 NCBI37). SAMtools [23] and BEDtools [24] were used for filtering steps and file format conversion. The peaks were identified from uniquely mapped reads without duplicates using MACS, and the *p*-value cutoff used for peak detection was 1 × 10^−5^. ChIP-seq data were normalized on DNA Input and expressed as a log2 value. ChIP-seq peaks were annotated with PAVIS [25]. IGV genome browser was used for data visualization (Figure 1). To plot data of average profiles around prime target genes, specific site positions were retrieved from the mouse genome (mm9).

### 2.7. Statistical Analysis

Statistical significance between two or three groups was assessed with Student’s *t*-test and or ANOVA test, respectively. Data are expressed as mean ± standard deviation (SD). All experiments were repeated at least three times. A *p*-value < 0.05 was considered statistically significant.

## 3. Results

### 3.1. RA induces Zscan4 Metastate and Prame Expression in ES^pZscan4-EME/PRAME-FLAG^

**Figure 2:**
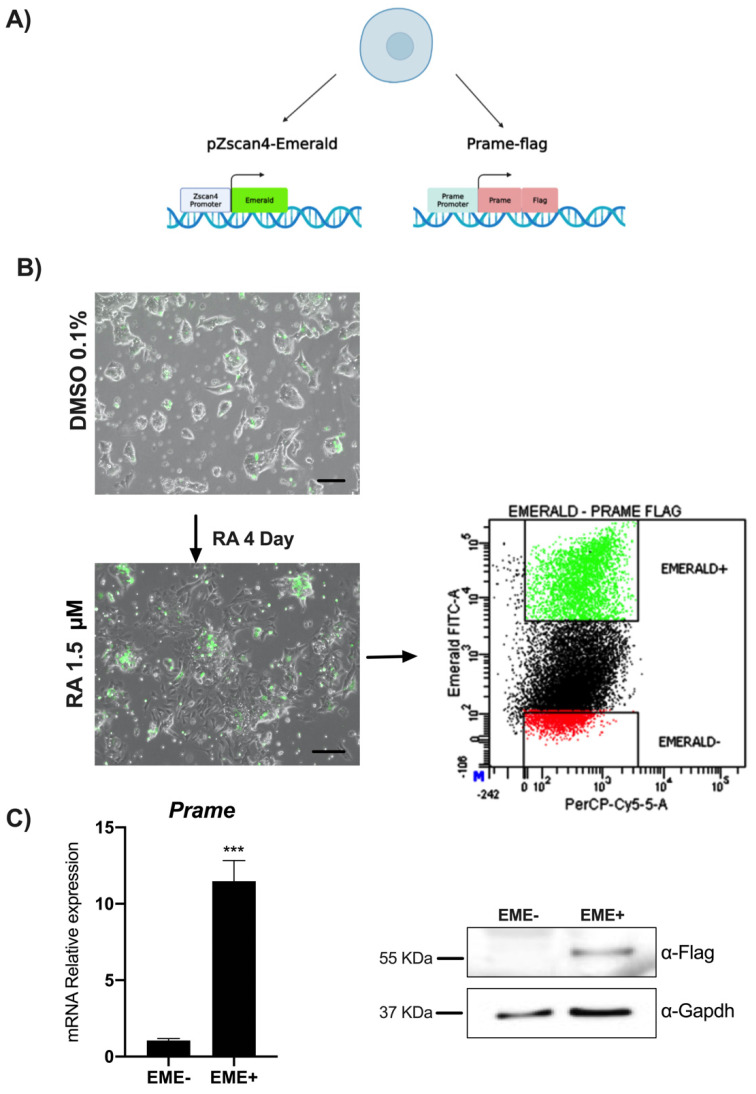
**RA induces Zscan4-Metastate and Prame expression in *ES^pZscan4-EME/Prame-FLAG^*.** (**A**) Schematic representation of ES transgenic cells for pZscan-Emerald and PRAME-FLAG expression. (**B**) *Z*scan4 and Prame positive cells visualized by Emerald reporter. After four days of RA treatment. Cells were imaged using phase-contrast microscopy (scale bars, 100 nm). Flow cytometry evaluation (dot blot) in *ES^pZscan4-EME/PRAME-FLAG^* cell lines of two subgroups, EME+ and EME-). (**C**) PRAME expression analysis for sorting validation by qPCR and western blot assays. Data are shown as mean ± SD from three independent experiments, normalized with Gapdh, and reported to the control (EME-). *** and *p* < 0.001.

To identify the Prame target genes, we used a transgenic ESCs line that simultaneously expresses FLAG-tagged Prame protein and Emerald downstream to the *Zscan4* promoter (ES*^pZscan4^*^-EME/Prame-FLAG^), as described in Material Methods and previously validated for expression of pluripotent marker genes [13]. ES*^pZscan4^*^-EME/Prame-FLAG^ cell line RA-treated was sorted for Emerald expression (or Zscan4 metastate), then immunoprecipitated with an anti-FLAG antibody and analyzed by next-generation sequencing (Figure 2A).

To obtain an enriched EME+ cell population, ES*^pZscan4^*^- EME/Prame-FLAG^ cells were cultured with 1.5 μM of RA for four days. Zscan4 induction was evaluated through fluorescence microscopy (Figure 2B) and FACS analysis to quantify Zscan4 expression; we analyzed the percentage of green cells (EME+) in ES*^pZscan4^*^- EME/Prame-FLAG^ that increased to 21.8% after RA treatment (Figure 2C). In addition, we further confirmed the successful separation of Prame gene and protein expression in EME+ cells by western blot (Figure 2C). Finally, we performed a ChIP-seq analysis in EME+ cells.

### 3.2. Identification of Prame Target Genes by ChIP-Seq Analysis

To identify Prame target genes upon RA treatment, we collected 2 × 10^6^ EME+ cells after sorting. In Figure S1A, we can see the distribution of Prame binding regions: upstream and downstream gene regions, 5′UTR and 3′UTR, introns in the form of a pie chart (Figure S1B). As shown in Figure S1B, binding is mainly found in regulatory regions (upstream, 3′-UTR and downstream gene regions).

The ChIP-seq analysis showed 15 specific genes significantly bound by Prame (*Cdk8*, *Cdkn2d*, *uc009gks.1*, *St6galnac1*, *Stard13*, *Mir26b*, *533042B09Rik*, *Gm15319*, *Gm3168*, *AK053193*, *Mir715*, *Zc3h7a*, *Filip1l*, *AK041614*, *and Gm10406*) and described in Table 2.

Among these, we chose *Cdk8* and *Cdkn2d* for further validation. Cdk8 is a cyclin-dependent kinase that maintains both tumors and embryonic stem cells in an undifferentiated state [16], while Cdkn2d (cyclin-dependent kinase inhibitor 2D) is a cell growth regulator that controls G1 cell cycle progression and results in dissymmetrically expressed cells in the 2-cell stage, thus, representing good targets of the pluripotency stage marked by Prame expression [14].

**Figure 3:**
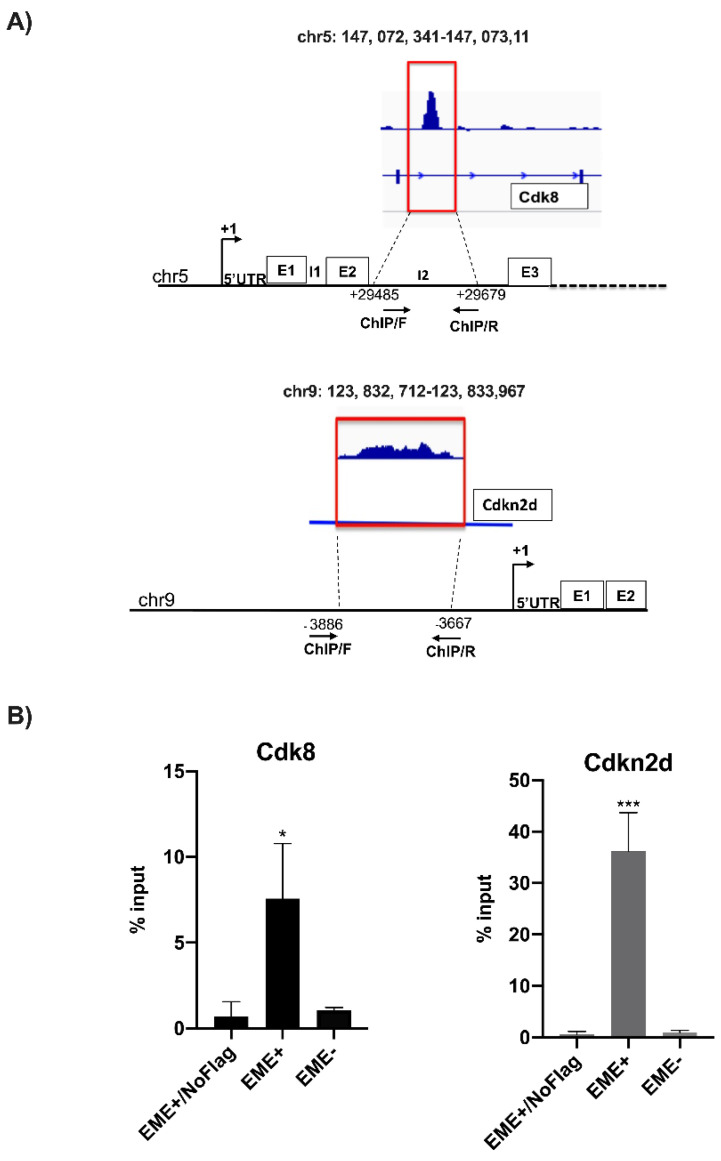
**Prame-target gene identification by ChIP-seq analysis and ChIP assay for validation.** (**A**) Schematic representations of *Cdk8* intron 2 and *Cdkn2d* upstream regions with enriched sequences, peaks were indicated on specific chromosome positions. (**B**) ChIP assay validation followed by qPCR analysis for Prame binding sites on *Cdk8* and *Cdk2d* gene sequences in EME+, EME, and EME+/NoFLAG cells (without Prame-FLAG) as the negative control. Data are shown as mean ± SD from three independent experiments, normalized with Gapdh, and reported to the control (EME-). *, *p* < 0.05; ***, and *p* < 0.001.

Figure 3A indicates the schematic position of primers used for ChIP analysis validation by qPCR on *Cdk8* and *Cdkn2d* enriched sequences after ChIP-seq.

EME+ and EME- cells and EME+/NoFLAG DNA immunoprecipitated for Prame, with anti-FLAG antibody, was analyzed through qPCR for *Cdk8* and *Cdkn2d* enrichment to confirm data obtained by ChIP-seq. Data are calculated as the percentage of the input (Figure 3B). The EME+/NoFLAG cells were used as a negative control of immunoprecipitation.

### 3.3. RA Induces Cdk8 and Cdkn2d Expression

**Figure 4:**
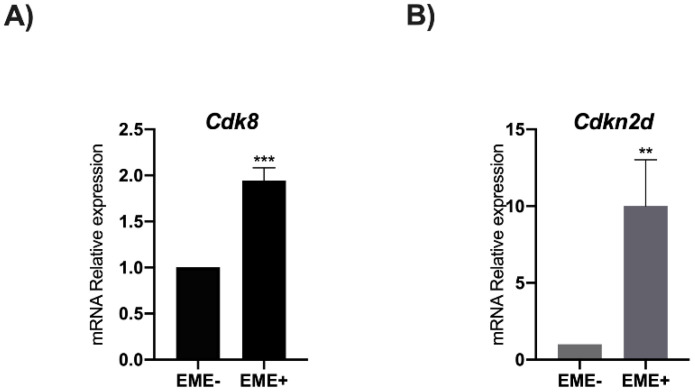
**RA induces *Cdk8* and *Cdkn2d* gene expression in ESCs.** (**A**) *Cdk8* and (**B**) *Cdkn2d* gene levels in EME+ cells after RA treatment. Data are shown as mean ± SD from three independent experiments, normalized with *Gapdh*, and reported to the control (EME-). **, *p* < 0.01 ***, and *p* < 0.001.

To understand how RA influences *Cdk8* and *Cdkn2d* expression, a qPCR analysis was performed in EME+ and in EME- cells (Figure 4A,B). The gene expression analysis showed a significant enhancement of *Cdk8* and *Cdkn2d* expression in EME+ cells compared to EME- (Figure 3). These data indicate that the Prame binding to Cdk8 and Cdkn2d genes increases their expression.

### 3.4. Prame Enhances Cdk8 and Cdkn2d Expression in ES^Gm12794cp2Lox^ Transgenic Cells

**Figure 5:**
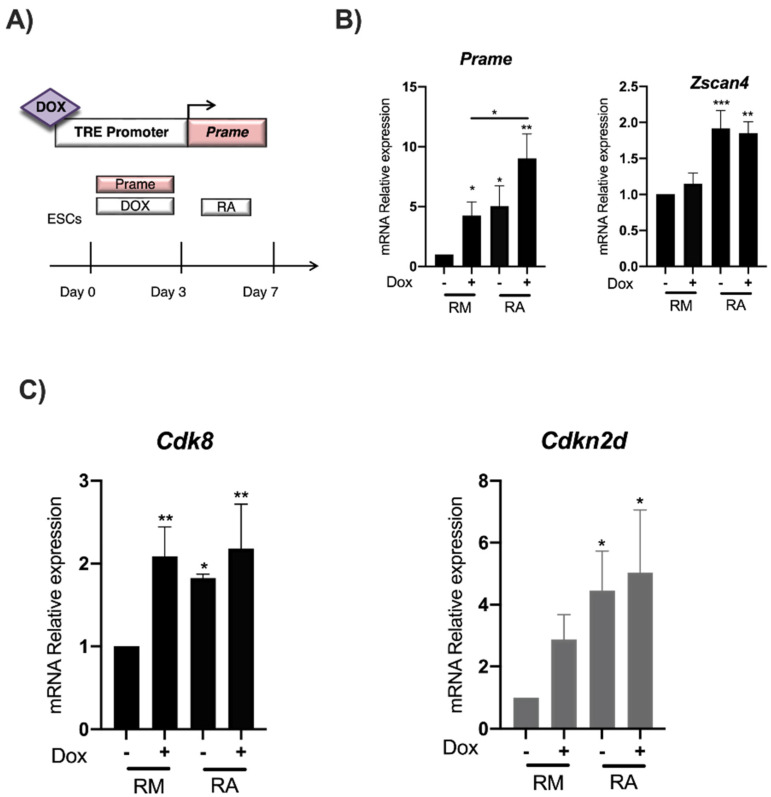
**Prame overexpression influences *Cdk8* gene expression in ES cells.** (**A**) Schematic representation of the minimal Dox-inducible promoter driving the expression of Prame in the ES^Gm12794cp2Lox^ cell line. (**B**) A qPCR analysis showing relative Prame and *Zscan4* mRNA levels in ES^Gm12794cp2Lox^ cells treated or not treated with Dox (1.5 μg/mL) for 72 h and then with RA (1.5 μM) for 4 days. (**C**) *Cdk8* gene expression, but not *Cdkn2d*, was induced directly by Prame overexpression in ES^Gm12794cp2Lox^ cells. (RM is the regular medium without RA). Data are shown as mean ± SD from three independent experiments, normalized with *Gapdh*, and reported to the control (Dox-). *, *p* < 0.05; **, *p* < 0.01 ***, and *p* < 0.001.

To understand whether Prame overexpression influences *Cdk8* and *Cdkn2d* gene expression in ES cells, an ES^Pramep2Lox^ cell line in which Prame expression is induced by doxycycline was employed [13]. The ESCs stably transfected with the inducible PRAME expressing construct were treated with doxycycline for 3 days (Figure 5A) and then with RA for 4 days. To validate Prame induction by doxycycline and then by RA treatment, both Prame and *Zscan4* gene expression were analyzed by qPCR (Figure 5B). The ES cell line p2Lox empty vector was used as a negative control for the Dox treatment. Our data shows a 5-fold higher *Prame* gene expression after the Dox treatment (Figure 5B), with cells maintaining a classic ESC pluripotent morphology as previously described [13]. *Zscan4* gene expression was observed after RA treatment, confirming the Zscan4 metastate induced by RA. Finally, we observed that *Cdk8* and *Cdkn2d* expressions were directly increased in ESCs overexpressing Prame without RA (Figure 5C). This data shows for the first time that Prame directly influences the expression of their target genes, also without RA treatment.

### 3.5. Prame Controls Cdk8 Expression

**Figure 6:**
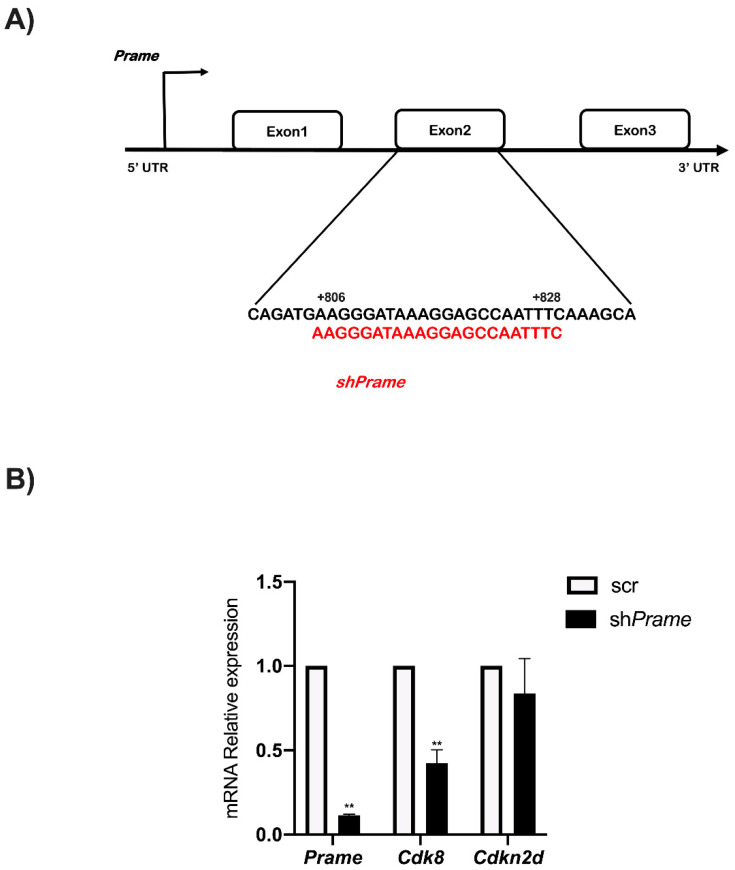
**The *Prame knock-down* specifically affects *Cdk8* gene expression in ESCs.** (**A**) Schematic representation of *shPrame* (sequence in red) localized in the second exon of *Prame* coding region at a distance indicated from TSS. (**B**) The ESC cells were transfected in transient for 24 h with *sh*Prame and scr (scrambled), the negative control. Data are shown as mean ± SD from three independent experiments, normalized with *Gapdh*, and reported with respect to the control. **, *p* < 0.01.

To shed light on the role of Prame in *Cdk8* and *Cdkn2d* gene regulation, transient inactivation of *Prame* was performed in E14 ES. The sh*Prame* vector and a sh-scrambled (sh-scr as a negative control) were transfected in ESCs for 24 h. *Prame* specific-shRNA mapped to the Exon2-coding region (Figure 6A). Total RNA was extracted from ES cells transfected with sh*Prame* and sh-scr. The qPCR analysis showed a significant reduction in *Cdk8* expression in ESCs upon *Prame* silencing compared to sh-scr cell control (Figure 6B), while *Cdkn2d* expression remains unaffected by *Prame* silencing (Figure 6B).

## 4. Discussion

The role of PRAME in human cancers is well established. PRAME is recruited in human cells to epigenetically and transcriptionally activate promoter regions bound by the nuclear transcription factor Y (NFY). This transcription factor is essential for early embryonic development [26] and has been implicated in the maintenance of the high proliferative capacity of ES cells [27] as well as in the inhibition of differentiation [28,29]. In addition, several studies indicated that Prame-like genes have roles in the early stages of spermatogenesis [30] and oogenesis [31], as well as in embryonic development and embryonic stem cells [32,33]. Moreover, Prame overexpression in E14 embryonic stem cells was reported to maintain a pluripotent state in the absence of the antidifferentiation factor LIF [13,34] and in ESCs that were RA-resistant [8].

Together, all these data are consistent with the notion that stem cells and neoplastic tissues share many properties, and several oncogenic pathways can also regulate self-renewal mechanisms in stem cells [35]. Therefore, the identification of Prame functions and targets indeed represents a step forward, not only in the cancer diagnostic approach but also in the therapeutic approach.

Here, we report, for the first time, two putative novel Prame targets in RA-treated ESCs. To better understand the regulatory network underpinning the establishment of naïve pluripotency marked by *Zscan4* and *Prame* genes in ESCs, we used *ES^pZscan4^*^-EME/Prame-FLAG^ transgenic cell lines, allowing us to immunoprecipitate Prame and its chromatin-bound fragments by using anti-FLAG antibodies. The Prame-bound sequences were characterized by ChIP-seq analysis. Among the putative Prame targets identified, we focused our attention on Cdk8, and Cdkn2d.

Cyclin-dependent kinases (CDKs) are key players in cell cycle regulation. Being involved in the regulation of cell cycle checkpoints, Cdk8, and Cdkn2d are involved in several cellular pathways, however, very little is known about their involvement in the establishment of RA-resistant Zscan4 metastate pluripotent cells. CDK8 was shown to maintain tumor dedifferentiation and embryonic stem cells pluripotency stage [16] drugs resistance in several cancer disease models [36,37]. Similar evidence was reported for Cdkn2d overexpression [38]. Concerning *Cdkn2d*, unlike *Cdk8*, we could not find any alteration in its expression upon Prame silencing. A possible explanation for this phenomenon is that Cdkn2d may be regulated by a higher expression level of Prame occurring under particular cell conditions.

Our data uncovered an important novel role of *Cdk8* and *Cdkn2d* expression in the Prame-dependent naïve pluripotent metastate. Remarkably, CDK inhibitors, such as CDK4/6 inhibitors, are already used in preclinical studies for cancer treatment [39]. In particular, CDK8 inhibitors have been used in acute myelogenous leukaemia and several other types of cancers, including breast cancer. Cancer cells treated with CDK8 inhibitors responded with decreased cell viability and increased apoptosis [40,41]. However, the function of Cdk8 and Cdkn2d may be cell-context dependent. Therefore, it will be necessary to deeply investigate the protein expression and the molecular and epigenetic mechanisms underlying Prame-induced Cdk8 and Cdkn2d activation in ESCs to identify the tumor specificity of anti-Cdk8 or anti-Cdkn2d pharmacological compounds affecting cancer cells mimicking the PRAME-marked stage of cell pluripotency.

## 5. Conclusions

In our work, we identified, for the first time, *Cdk8* and *Cdkn2d* as new Prame -target genes through a ChIP-seq analysis specific to ESCs enriched in Prame expression after RA treatment.

The identification of new targets for Prame in ESCs represents a milestone in the field of ESC therapy, specifically for CSC drug delivery. Our data opens a new window on the study of CSC therapies, RA-resistant. In the future, it will be necessary to use drugs and combinations of these in cancers marked by *Cdk8* and *Cdkn2d* expression.

## Figures and Tables

**Table 1:** List of genes and sequences of primers used for relative gene expression analysis by qPCR.

Gene	Sequence Fw (5′-3′)	Sequence Rv (5′-3′)	^1^ Method
*Cdkn2d*	CCACCGGTATCCACTATGCT	GCACAGGACTAGTACCGGAG	qPRC
*Cdk8*	TCCAGTGGTTGTAACATTCTGGT	TTCTGCAAATATACACCCTATAGCC	qPRC
*Prame*	TGCTGCCAAATTCCTTTCTC	GAGAGTTGGCAGCGATTCAT	qPRC
*Gapdh*	AATGGTGAAGGTCGGTGTG	GAAGATGGTGATGGGCTTCC	qPRC
*Cdkn2d*	TCCTCATGCTGGTTCTGTGT	AGGAGGGAAAACAAGGGCTT	ChIP
*Cdk8*	TTAAATGAGACATGGGCGCG	GGAGTTTACCACCCGCTTTG	ChIP

^1^ In the Method column, qPCR indicates the gene expression analysis, while primers used for ChIP assay are indicated in the Method column with ChIP.

**Table 2:** Prame target genes identified by ChIP-seq analysis in EME+ cell lines.

Gene	Category	Chr	Description
*Cdk8*	Intron	5	Cell division protein kinase 8
*Cdkn2d*	Upstream, Downstream	9	Cyclin-dependent kinase 4 inhibitor D
*uc009gks.1*	Upstream, Intron, 3′UTR, Downstream	7	Hypothetical protein LOC72244
*St6galnac1*	Intron	11	α-N-Acetyl-galactosaminide
*Stard13*	Intron	5	stAR-related lipid transfer protein 13 isoform
*Mir26b*	Downstream	1	Mus musculus microRNA 26 b (Mir26b), microRNA
*5330429B09Rik*	Downstream	8	Subname: Full = putative uncharacterized protein
*Gm15319*	Intron	8	Testis-specific gene with ankyrin repeats
*Gm3168*	3′UTR	8	Subname: Full = Gag protein
*AK053193*	Downstream	10	Mus musculus 9,5 days embryo parthenogenote cDNA, RIKEN full
*Mir715*	Downstream	17	Mus musculus microRNA 715 (Mir715), microRNA
*Zc3h7a*	Intron	16	Zinc Finger CCCH-type containing 7A
*Filip1l*	Intron	16	Filamin A-interacting protein 1-like isoform 1
*AK041614*	Downstream	15	Mus musculus 3 days neonate thymus cDNA, RIKEN full-length enriched library
*Gm10406*	Intron	14	Alpha7-takusan

## Data Availability

Not applicable.

## Supplementary Materials

The following supporting information can be downloaded at: https://www.mdpi.com/article/10.3390/genes13101745/s1, Figure S1: The enriched regions after anti-FLAG antibody immunoprecipitation.
