# Supplementary material for: Identification of Cdk8 and Cdkn2d as New Prame-Target Genes in 2C-like Embryonic Stem Cells
Source: Genes (Basel). 2022 Sep 27;13(10):1745. doi: 10.3390/genes13101745 (PMC9601988; doi:10.3390/genes13101745)

Figure S1. (A) The enriched regions after anti-Flag antibody immunoprecipitation are indicated in the table after the UCSC genome browser analysis. **(B)** Pie chart showing the distribution of all binding sites for Prame.

A)

Location	Query Peak	Number	Proportion	EnrichTest1	EnrichTest2	Comparison Peak
Upstream	Q-Upstream	4	6,7%	9,59E-01	6,45E-01	C-Upstream
5' UTR	Q-5UTR	0	0,0%	1,00E+00	1,00E+00	C-5UTR
Exons/CDS	Q-Exon	1	1,7%	8,68E-01	6,71E-01	C-Exon
Introns	Q-Intron	23	38,3%	NA	9,59E-01	C-Intron
3' UTR	Q-3UTR	6	10,0%	3,57E-03	1,69E-04	C-3UTR
Downstream	Q-Downstream	9	15,0%	4,14E-01	3,69E-02	C-Downstream
Unclassified	NA	17	28,3%	NA	NA	NA

B)

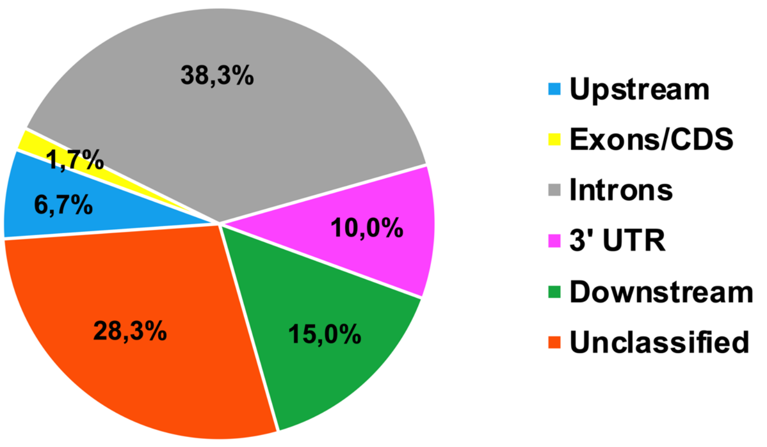

Supplement: Supplementary file 1 [file genes-13-01745-s001.zip › genes-1877783-supplementary.pdf]
